# Patient Safety of Perioperative Medication Through the Lens of Digital Health and Artificial Intelligence

**DOI:** 10.2196/34453

**Published:** 2023-05-31

**Authors:** Jiancheng Ye

**Affiliations:** 1 Feinberg School of Medicine Northwestern University Chicago, IL United States

**Keywords:** perioperative medicine, patient safety, anesthesiology, human factors, medication errors, digital health, health information technology

## Abstract

Perioperative medication has made significant contributions to enhancing patient safety. Nevertheless, administering medication during this period still poses considerable safety concerns, with many errors being detected only after causing significant physiological disturbances. The intricacy of medication administration in the perioperative setting poses specific challenges to patient safety. To address these challenges, implementing potential strategies and interventions is critical. One such strategy is raising awareness and revising educational curricula regarding drug safety in the operating room. Another crucial strategy is recognizing the importance of redundancy and multiple checks in the operating room as a hallmark of medication safety, which is not a common practice. Digital health technologies and artificial intelligence (AI) also offer the potential to improve perioperative medication safety. Computerized physician order entry systems, electronic medication administration records, and barcode medication administration systems have been proven to reduce medication errors and improve patient safety. By implementing these strategies and interventions, health care professionals can enhance the safety of perioperative medication administration and improve patient outcomes.

## Introduction

Medication errors represent a critical patient safety problem, arising from failures in completing required actions or using the wrong plan or action to achieve patient care aims [1]. These errors can be classified by type, including incorrect dose, substitution, omission, repetition, insertion, and unattended drug use [2]. In the perioperative setting, the administration of intravenous (IV) medications, or the “medication use process” [3], presents unique patient safety obstacles. The medication use process consists of several steps, including requesting, dispensing, preparing, administering, documenting, and monitoring patients for the effects of medication [4]. However, compared to almost any other hospital setting, medication administration in the operating room lacks most standard and accepted safety checks [1]. Unlike nurses, who require a physician to place an order for a medication, the pharmacy to prepare the medication, and a second nurse to verify the medication prior to administration, the anesthesiologist working alone in the operating room can determine the need for a medication (ie, diagnose and prescribe), draw up the medication (ie, prepare), administer and monitor the effects of the medication, and record events without any verification check for safety and accuracy. Furthermore, the often fast-paced and high-stress environment of the operating room can further increase the likelihood of medication errors. Thus, it is not surprising that medication errors are common in this setting. To address this issue, it is essential to implement strategies and interventions that improve the safety and accuracy of the process of medication use in the operating room.

### Patient Safety Problems Associated With Perioperative Medication Errors

Medication errors can be classified based on their potential for patient harm and whether they result in an adverse drug event or not. These classifications include errors with no potential for harm (near miss), those with little potential for patient harm, those with potential for adverse drug events, and those resulting in adverse drug events [1]. An adverse drug event is defined as any patient injury resulting from medication [5]. However, it is essential to note that adverse drug events can occur even without medication errors; for example, in the case of an allergic reaction. Nanji et al [1] further classified medication errors and adverse drug events by their severity (significant, serious, life-threatening, and fatal) and preventability (definitely preventable, probably preventable, probably not preventable, and definitely not preventable), and they found that out of the 193 (of a total of 3671, 5.3%) identified medication errors and adverse drug events, 153 (79.3%) were preventable. Additionally, 32 (20.9%) of these medication errors had little potential for harm, 70 (45.8%) had the potential for patient harm, and 51 (33.3%) resulted in an adverse drug event. The errors were further classified as serious (n=99, 64.7%), significant (n=51, 33.3%), and life-threatening (n=3, 2%), with no fatalities attributable to medication errors. In a separate study by Cooper et al [2], the authors identified 52 medication errors, resulting in no harm in 24 patients, minor harm in 15 patients, and harm in 13 patients.

### Risk Factors

Medication errors have been a concern since the 1970s, but the exact prevalence of these errors is still unknown due to underreporting [3]. According to Cooper et al [6], human factors are the primary cause of medication errors. These factors include failure to check, poor labeling, syringe swaps, decreased vigilance, fatigue, and production pressure. Distraction, pressure to proceed, and misread labels were found to be the top 3 factors contributing to medication errors. Nanji et al [1] identified the following as the 3 most common medication errors: wrong dose, improper labeling, and failure to deliver the appropriate medication. High-acuity medications such as propofol, phenylephrine, and fentanyl were the most common perioperative medications involved in these errors [7].

Despite increased awareness and emphasis on perioperative medication safety by the Anesthesia Patient Safety Foundation, little progress has been made in addressing the human factor constraints that lead to medication errors. These constraints include lack of standardized labels; varied drug vial sizes, shapes, and colors; poorly designed medication carts and drug dispensing machines; and look-alike and sound-alike medications. The labeling and packaging of medications were found to be contributors to almost one-third of voluntarily reported medication errors leading to fatalities in the 1990s, according to the Institute of Safe Medication Practices’ Medication Errors Reporting Program [8]. The Food and Drug Administration estimates that suboptimal labeling and packaging contribute to approximately 20% of medication errors. Moreover, drug shortages may force pharmacies to source unfamiliar substitute medications that can lead to errors, as reported by 21% of hospital pharmacists in a 2017 survey [9].

The high rate of medication errors in perioperative medication administration can be attributed to several factors, but perhaps the most significant is the anesthesia culture itself. The norm of an anesthesiologist performing the medication administration process with no oversight is deeply ingrained in professional and organizational culture. Prielipp et al [10] discussed the concept of “normalized deviance” in anesthesia practice, where departure from correct behavior becomes so ingrained in work culture that it is no longer considered deviant. Despite evidence that independent double checks can detect up to 95% of potential medication errors and eliminate 58% of those identified [11], the culture of autonomy, rejection of “cookbook” medicine, and resistance to standardization hinder the reporting of minor or near-miss events and impede efforts to improve medication safety in anesthesia. Grigg and Roesler [3] summarized this culture as allowing providers to “hand scrawl on poorly labeled syringes drawn up from nonstandard, look-alike vials in a distracting environment and organize them in an arbitrary, personalized arrangement.“ Organizational barriers also exist, such as fear of reprisal for errors or a blame culture, which can prevent medication errors from being reported [12].

### Interventions and Recommendations

Inadequate labeling and packaging of medications is one of the primary human factors associated with a significant number of medication errors. Poor labeling can result in the administration of the wrong medication, incorrect dosage, and an incorrect route of administration. Common labeling errors include syringes that are unlabeled or inaccurately labeled and illegible, handwritten labels [13]. To address the labeling problem, various solutions have been proposed. These include using standardized color-coded labels for similar drug classes, implementing barcode-assisted labeling systems to generate medication labels, and using commercially prepared prefilled medication syringes. In a systematic review by Maximous et al [14], improved labeling led to a reduction of 37% in medication errors. Recent data have questioned the effectiveness of color-coding in preventing medication errors [15], but research on human factors engineering (HFE) highlights the importance of pattern recognition when performing pressured, high-stress tasks, such as administering high-risk medication in the operating room environment [6]. Although support for the color-coded labeling system has somewhat decreased, HFE acknowledges that identifying objects in high-stress situations relies on multiple cues [3], and the historical use of color-coding remains a crucial cue for anesthesiologists.

Digital health technologies such as computerized physician order entry (CPOE) systems and electronic medication administration records (eMAR) have been shown to reduce medication errors and improve patient safety. CPOE systems allow health care professionals to electronically enter medication orders, reducing the likelihood of errors due to illegible handwriting or transcription errors. The use of CPOE systems can ensure that medication orders are accurately and efficiently transmitted to the pharmacy and the surgical team. The eMAR systems can provide real-time information about medication administration during surgery, allowing the surgical team to make informed decisions about the patient's care [16]. eMAR reduces the risk of medication errors due to incorrect dosages or administration times. In the perioperative setting, these digital health technologies can be particularly beneficial.

Barcode medication administration (BCMA) technology has been successful on nursing floors, but is not yet widely implemented in anesthesia due to the cost of the systems and a lack of a universal electronic health record (EHR) system capable of scanning barcodes and incorporating the information into the operative record [17]. BCMA systems use barcodes to verify patient and medication information, thus reducing the risk of errors due to incorrect medication selection or administration. BCMA systems can help ensure that the correct medications are administered to the correct patient at the correct time, reducing the risk of medication errors and adverse events. Point-of-care barcode scanning has the potential to eliminate 17% of medication errors and 25.5% of potential adverse drug events [1]. In a study by Merry et al [18], a 21% reduction in perioperative medication errors was demonstrated when syringe labels were scanned immediately before administration. When automated drug-specific decision support and alerts were added, an additional 29% and 59% of medication errors could be eliminated, respectively. Despite the availability of barcode-assisted labeling systems in their study environment, the authors found that up to 24% of medication errors still involved a labeling error. These errors occurred when health care professionals bypassed the system or found a workaround. To minimize these risks, prefilled medication syringes prepared at standard concentrations and provided by the pharmacy may be the best risk reduction strategy. A failure modes and effects analysis of the use of prefilled syringes has the potential to eliminate 16 medication preparation steps and 19 potential failure modes [19]. For organizations with barcode scanning abilities, these prefilled syringes could also use barcode technology, which would eliminate compounding of medications by the anesthesia clinicians and the associated risks of this practice. Other process-based interventions, such as facilitating timing of documentation, reducing workarounds, and standardizing connections of IV drug infusions to the most proximal port, could further reduce medication errors. These process interventions have the potential to eliminate 35%, 24%, and 1.3% of medication errors, respectively [1]. Ultimately, multimodal strategies are needed, which include all potential human factor system changes and process interventions discussed above. Multimodal interventions, including barcode readers with automatic auditory and visual verification of the drug, prefilled color-coded syringes, and workspace improvements including standardized stocking of anesthesia carts, have the greatest potential to reduce errors [20]. In combination, these interventions could reduce error rates by 21%-35% per administration and 37%-41% per anesthetic [14].

Artificial intelligence (AI) has the potential to revolutionize medication safety by providing real-time decision support, reducing medication errors, and improving communication among health care professionals [21]. AI can analyze large data sets to identify patterns and predict adverse events, allowing health care professionals to intervene before harm occurs. AI can also provide decision support, suggesting the most appropriate medication and dosage for a particular patient based on their medical history and other factors. Finally, AI can facilitate communication among health care professionals, ensuring that critical information is shared in a timely and efficient manner. In the perioperative setting, AI can be particularly valuable in predicting and preventing adverse events. For example, AI algorithms can analyze vital signs and other patient data to identify patients at risk for postoperative complications such as sepsis or acute kidney injury [22]. AI can also provide decision support to help health care professionals select the most appropriate medication and dosage for a particular patient, taking into account their medical history, allergies, and other factors. Finally, AI can facilitate communication among health care professionals, ensuring that critical information is shared in a timely and efficient manner.

AI algorithms can analyze large amounts of data to identify patterns and predict medication errors. For example, AI can analyze medication orders and patient data to identify patients at high risk for medication errors. This can help clinicians to proactively intervene to prevent errors before they occur. Natural language processing algorithms can analyze free-text notes in the EHR to identify potential medication errors. For example, natural language processing can identify notes that mention medication errors or adverse drug events. This can help clinicians to identify and address medication errors that may have been missed through other means. AI can be used to provide real-time decision support to clinicians. For example, AI algorithms can analyze medication orders and provide alerts to clinicians about potential drug interactions, dosing errors, or other safety concerns. This can help clinicians to make informed decisions about medication orders and reduce the risk of errors. Table 1 outlines specific ways that AI can be used to improve perioperative medication errors.

**Table 1:** Application of artificial intelligence (AI) in the patient safety of perioperative medication.

AI technology	Applications
Predictive analytics	AI algorithms have the potential to analyze vast amounts of data, identify patterns, and predict medication errors [23]. For example, clinicians can proactively intervene to prevent errors before they occur by analyzing medication orders and patient data to identify patients at high risk for medication errors, using AI.
Natural language processing	Natural language processing algorithms can analyze free-text notes in the electronic health record to identify potential medication errors that may have been missed through other means, such as notes that mention medication errors or adverse drug events [24]. This approach can assist clinicians in identifying and addressing potential errors before they cause harm.
Clinical decision support	AI can provide real-time decision support to clinicians by analyzing medication orders and providing alerts for potential drug interactions, dosing errors, or other safety concerns. This feature can assist clinicians in making informed decisions about medication orders and reducing the risk of errors.
Machine learning	Machine learning algorithms can be used to identify patterns and predict medication errors by analyzing medication orders and patient data. These algorithms can also be used to develop personalized medication regimens for individual patients based on their unique characteristics, which can improve medication safety and reduce the risk of adverse drug events [25].
Computer vision	Computer vision algorithms can be used with barcoding systems to verify medication administration [18]. For instance, computer vision can analyze barcode scans to verify that the medication matches the order and the patient's information in the electronic health record. This feature can help reduce the risk of errors due to incorrect medication administration.

## Discussion

Ensuring patient safety is a paramount concern, especially when it comes to administering medications in the perioperative setting. Medication reconciliation is a crucial process that involves comparing a patient's medication orders with their current medication regimen to identify any discrepancies and prevent medication errors caused by incomplete or inaccurate medication histories. To ensure medication safety, it is important to perform preoperative medication reconciliation and document it accurately [26]. Standardized protocols for medication administration, such as those recommended by professional societies or institutions, can help reduce the risk of errors during drug preparation, dosage calculation, and administration [27]. Education and training are essential for improving the safety of perioperative medication. Clinicians should be trained on medication safety best practices, including the use of decision support tools, the importance of medication reconciliation, and the use of standardized protocols [28]. Effective communication among health care professionals, especially during handovers, is critical to reducing medication errors. The use of standardized communication tools and training can help improve communication among health care professionals [29]. Continuous electronic monitoring of vital signs, particularly during surgery, can help identify and manage medication-related adverse events promptly. Emerging evidence suggests that incorporating HFE principles into practice may improve patient safety by reducing cognitive workload and simplifying medication administration processes [30]. By optimizing the design of medication administration processes, we can reduce the risk of errors and improve patient safety [31].

Digital health technologies and AI can be used to enhance perioperative medication safety by detecting potential medication errors and providing decision support tools to clinicians in real time. Clinical decision support tools, such as alerts for potential drug interactions or incorrect doses, can help to prevent medication errors [32]. These tools can be integrated into EHR systems, providing clinicians with accurate and up-to-date information about medications and their effects. CPOE systems allow clinicians to enter medication orders directly into an EHR system, reducing errors caused by illegible handwriting or transcription errors [33]. These systems can also provide decision support tools, such as alerts for potential drug interactions or incorrect doses. Additionally, CPOE systems can help to standardize medication orders, reducing the risk of errors due to miscommunication or confusion. The eMAR systems allow health care professionals to electronically record medication administration, reducing errors due to incorrect dosages or administration times [34]. These systems can also be integrated with barcode scanning technology to ensure that the correct medication is administered to the correct patient at the correct time. Telepharmacy services can be used to provide medication-related support to health care professionals in remote or underserved areas, ensuring that medication orders are accurate and complete and that medications are administered safely [35]. Patient portals can be used to provide patients with information about their medications, including dosages, side effects, and potential interactions [36]. These portals can also be used to remind patients to take their medications and to provide instructions on how to properly administer medications. By incorporating these digital health technologies and AI, health care professionals can reduce the risk of medication errors and improve patient outcomes.

Improving patient safety during perioperative medication administration requires a multifaceted approach, incorporating strategies that address medication reconciliation, standardized protocols, effective communication, continuous monitoring, and HFE principles.

## Conclusions

Perioperative medication safety has been largely overlooked in terms of rigorous assessment of medication events and the implementation of safety measures. Unlike other high-risk industries, there are few safety protocols to prevent simple but dangerous medication errors, such as those related to labeling. Addressing systemic weaknesses that contribute to medication errors requires HFE and cultural reforms, and a shift away from focusing on individual blame and failure of truth-telling and transparency to enable real reform. Simply improving vigilance is insufficient since it does not address human factors and systemic issues that contribute to errors. Digital health technologies and AI offer significant promise in enhancing perioperative medication safety. Systems such as CPOE, eMAR, and BCMA can reduce medication errors and improve communication among health care professionals. AI can provide real-time decision support, predict adverse events, and facilitate communication. However, it is necessary to develop effective ways to measure medication errors and capture data to identify the true scope of the problem and develop solutions for mitigation. Standardization, medication reconciliation, education and training, clinical decision support, barcoding and electronic medication administration, and effective team communication are all crucial to improving perioperative medication safety. By implementing these strategies, health care professionals can reduce the risk of medication errors and improve patient safety.

## Abbreviations

AI: artificial intelligence
BCMA: barcode medication administration
CPOE: computerized physician order entry
EHR: electronic health record
eMAR: electronic medication administration record
HFE: human factors engineering
IV: intravenous
