# Correction: Patient Safety of Perioperative Medication Through the Lens of Digital Health and Artificial Intelligence

**DOI:** 10.2196/84392

**Published:** 2025-10-08

Following the publication of “Patient Safety of Perioperative Medication Through the Lens of Digital Health and Artificial Intelligence” [1], concerns were raised regarding the corresponding author Jiancheng Ye’s rate of self-citation within the article’s reference list. Further investigation into this matter identified that numerous self-citations were added to the manuscript following acceptance of the article without adequate disclosure.

The author was contacted for a response regarding this matter. The author indicated that the added references were pertinent to the article and its findings, and provided documentation justifying the inclusion of the identified self-citations.

Following review of this response, the JMIR Publications Editorial Office determined that several of the added references were not relevant to the article or were used to support a generic statement where another reference could have been used instead of self-citation. Therefore, the JMIR Publications Editorial Office is issuing this corrigendum to remove the following references:

- Reference 5: Feinglass J, Wang JA, Ye J, Tessier R, Kim H. Hospital care for opioid use in Illinois, 2016-2019. J Behav Health Serv Res 2021 Oct;48(4):597-609 [doi: 10.1007/s11414-020-09748-8] [Medline: 33502670]
- Reference 6: Ye J. Health information system’s responses to COVID-19 pandemic in China: a national cross-sectional study. Appl Clin Inform 2021 Mar 19;12(2):399-406 [doi: 10.1055/s-0041-1728770] [Medline: 34010976]
- Reference 19: Ye J, Orji IA, Baldridge AS, Ojo TM, Shedul G, Ugwuneji EN, Hypertension Treatment in Nigeria Program Investigators. Characteristics and patterns of retention in hypertension care in primary care settings from the Hypertension Treatment in Nigeria program. JAMA Netw Open 2022 Sep 01;5(9):e2230025 [doi: 10.1001/jamanetworkopen.2022.30025] [Medline: 36066896]
- Reference 21: Ye J, Ren Z. Examining the impact of sex differences and the COVID-19 pandemic on health and health care: findings from a national cross-sectional study. JAMIA Open 2022 Oct;5(3):ooac076 [doi: 10.1093/jamiaopen/ooac076] [Medline: 36177395]
- Reference 24: Ye J, Ma Q. The effects and patterns among mobile health, social determinants, and physical activity: a nationally representative cross-sectional study. AMIA Jt Summits Transl Sci Proc 2021;2021:653-662 [Medline: 34457181]
- Reference 30: Li Q, Fu R, Zhang J, Wang R, Ye J, Xue N, et al. Label-free method using a weighted-phase algorithm to quantitate nanoscale interactions between molecules on DNA microarrays. Anal Chem 2017 Mar 21;89(6):3501-3507 [doi: 10.1021/acs.analchem.6b04596] [Medline: 28230978]

The JMIR Publications Editorial Office regrets that these issues were not identified prior to publication.

The correction will appear in the online version of the paper on the JMIR Publications website together with the publication of this correction notice. Because this was made after submission to PubMed, PubMed Central, and other full-text repositories, the corrected article has also been resubmitted to those repositories.
